# Autologous internal limiting membrane transplantation achieves anatomic closure and functional improvement in the treatment of large, persistent macular holes

**DOI:** 10.1186/s40942-023-00524-2

**Published:** 2024-01-18

**Authors:** Hanna Camenzind-Zuche, Lucas Janeschitz-Kriegl, Pascal W. Hasler, Christian Prünte

**Affiliations:** 1 Department of Ophthalmology, University Hospital Basel, University of Basel, Basel, Switzerland; 2 Institute of Molecular and Clinical Ophthalmology Basel (IOB), Basel, Switzerland

**Keywords:** Persistent full-thickness macular hole, Small-incision vitrectomy, Internal limiting membrane transplantation

## Abstract

**Purpose:**

To evaluate the clinical outcome of subretinal autologous internal limiting membrane (ILM) transplantation during pars-plana vitrectomy for persistent full-thickness macular hole (FTMH) repair.

**Methods:**

Retrospective, consecutive case series of 13 eyes (13 patients) undergoing small-incision vitrectomy with ILM transplantation and air tamponade for large persistent FTMH after prior unsuccessful vitrectomy with posterior hyaloid detachment and ILM peeling.

**Main outcome measurements:**

For all eyes, high-definition spectral domain optical coherence tomography scans (SD-OCT Spectralis, Heidelberg Engineering GmbH, Germany) of the macula were routinely performed before surgery, 1 and 4 weeks after surgery, and at the final follow-up visit. Additionally, age, gender, axial length, macular hole diameter, biomicroscopic fundus evaluation and best‐corrected visual acuity (BCVA) at baseline, 1 and 4 weeks after surgery, and at the final follow-up visit were analyzed.

**Results:**

Anatomic closure was achieved in all 13 cases (100% success rate). Closure pattern was classified in accordance with to Rossi et al. (Graefe’s Arch Clin Exp Ophthalmol 258(12):2629–2638, 2020). Mean baseline BCVA logMAR was 0.93, mean postoperative BCVA logMAR was 0.66 with a mean postoperative follow-up period of 11.4 months. No re-opening occurred during the observation period.

**Conclusions:**

Placing an autologous ILM-transplant in the subretinal space beneath the margin of the FTMH can support anatomic restauration and functional improvement in large, persistent FTMHs.

**Supplementary Information:**

The online version contains supplementary material available at 10.1186/s40942-023-00524-2.

## Background

A macular hole (MH) is a vitreoretinal interface disease characterized by a full-thickness defect in the central macular [1]. Pars plana vitrectomy (PPV) with posterior hyaloid detachment and internal limiting membrane (ILM) peeling is the current state-of-the-art surgical treatment for primary idiopathic full-thickness macular holes (FTMH), with reported closure rates of as high as 91–98% [2–6]. Despite the success of combining vitrectomy with ILM peeling, Ip et al. reported a 7–44% failure rate to restore anatomical and functional integrity, particularly in large FTMH greater than 500 µm [7, 8].

In addition to the hole diameter, several other factors contributing to failed closure have been described. These include chronic MH, persisting epiretinal traction, insufficient gas tamponade or poor patient compliance with maintaining a prone position [3, 9]. Further challenging situations may include high myopia, post-traumatic MH, chronic MH with persistence longer than one year or refractory MH that had one or more failed surgeries before [10].

Retinal surgeons developed multiple surgery techniques to tackle refractory FTMH. Most attention was attained for repeated vitrectomy with autologous transplantation of ILM [11], amniotic membrane transplant [12, 13] or autologous retinal transplant [14]. Other techniques include repeated PPV with ILM re-peeling and endotamponade with long-lasting gas [15], membranectomy and autologous serum [16], lens capsular flap transplantation [17, 18], induction of macular detachment with a balanced salt solution (BSS) [19–21], silicone oil tamponade [22], autologous platelet concentrate [23, 24] and radial retinal incisions [25–27].

However, only non-randomized and non-controlled studies with various success rates and without definitive management recommendations have been reported so far.

The reported techniques in the literature include covering the MH or plugging the ILM transplant into the MH [11, 28–30]. In this article, we describe a refinement of the method of autologous ILM transplantation. Our technique employs an autologous ILM transplant that is positioned subretinally beneath the edges of the hole, serving as a guiding scaffold for cellular migration and/or proliferation. We present a case series of 13 eyes treated successfully with this novel method to achieve anatomical and functional repair in persisting FTMH.

## Methods

### Study participants

In this retrospective, consecutive case series, closure rate, BCVA Snellen and BCVA logMar were evaluated in eyes with persistent macular hole after 1 or 2 unsuccessful vitrectomies including detachment of the posterior vitreous, ILM peeling and air endotamponade. Small incision re-vitrectomy, peeling of remaining ILM and autologous ILM transplantation with subretinal spreading underneath the macular hole followed by air-tamponade, was performed.

Inclusion criteria were 1 or 2 previously unsuccessful surgeries for FTMH. Good quality OCT scans at baseline, at 4 weeks and at the last follow-up visit for all eyes were analyzed.

### Data collection

The following parameters were retrieved from the patient files: age, gender, sex, axial length, macular hole diameter on SD-OCT, previous surgeries, observational period and best‐corrected visual acuity (BCVA) in logMAR and Snellen, baseline and postoperative at 1 and 4 weeks and at the last follow-up visit (mean 11.4 months).

All patients underwent an extensive eye examination, including best-corrected visual acuity (BCVA) testing, dilated fundus examination with slit-lamp biomicroscopy and OCT at baseline, at 4 weeks and at the last follow-up. The FTMHs were grouped on the basis of classification proposed by the IVTS group [31].

### Spectral domain OCT analysis

Based on a detailed analysis of the patients’ SD‐OCT images, the FTMHs were classified in clinical stages according to the IVTS Group classification system. The FTMH mid-length diameter (MLD) was measured by SD‐OCT (OCT Spectralis®, Heidelberg Engineering GmbH, Germany) using the caliper function. The MLD was measured at the narrowest aperture of the FTMH.

The closure of a FTMH was defined as can be seen in primary macular hole surgery with re-adaptation of the neurosensory retinal tissue at the rim of the macular hole. A definition of macular hole closure type was done according to Rossi et al. [32]. In this case series, successful closure of the macular hole was Type 1. Atrophy occurred in all patients with type O classification.

### Surgical procedure

Small-incision three-port 25-gauge vitrectomy was performed in peribulbar anaesthesia in all patients by one experienced eye-surgeon (CP). Residual ILM was stained with Brilliant Blue G: 0.125 mg (0.25 g/L; ILM-BLUE® D.O.R.C, The Netherlands) and a round patch of ILM extending the diameter of the macular hole was peeled in the peripheral macula. This ILM flap was placed into the macular hole and then spread under the rims of the hole with a bland spatula. Surgery was completed by fluid-air exchange (Additional file 1: Video S1). All patients were advised to maintain face-down positioning during the first 3 days after surgery.

### Statistical analysis

Statistical analyses, including descriptive statistics, were performed for all outcome measures. Results are reported as means and standard deviations, if not stated otherwise. Normal distribution of the data was tested using the Kolmogorov–Smirnov test. Depending on the distribution, either parametric tests (paired t-test) or non-parametric tests (Wilcoxon matched-pairs signed-rank test) were applied to compare the preoperative and postoperative data. The significance level was set at 0.05.

For the statistical analyses, MedCalc Statistical Software version 19.6 (MedCalc Software bv, Ostend, Belgium; https://www.medcalc.org; 2020) was used. Figures were created using GraphPad Prism 9.3.1 (GraphPad Prism Software, Boston; https://www.graphpad.com).

All patients signed an informed consent; the study was adherent to the Declaration of Helsinki and was approved by the local ethics committee.

## Results

A total of 13 patients, comprising 3 males and 10 females ranging from 53–83 years (mean ± SD: 69 ± 8 years), underwent vitrectomy with autologous submacular ILM transplantation. All patients had a FTMH hole, which persisted after a first or second vitrectomy with posterior hyaloid detachment and ILM peeling during the first surgery. The pre-operative size of the FTMH ranged from 418–830 μm (mean ± SD: 633.08 ± 119.02, Tables 1, 2) and the preoperative axial length ranged from 22.03–33.39 mm (mean ± SD: 27 ± 3.4, Table 1).

**Table 1:** Mean baseline characteristics of the patients and BCVA logMAR pre- and postoperative

	Age	Axial length (mm)	Foramen diameter (µm)	BCVA logMAR at baseline	BCVA logMAR post surgery
Mean	68.75	27.0	616.67	0.92	0.66
SD	8.38	3.4	107.86	0.26	0.45

**Table 2:** Individual patients’ characteristics

Patient	Age (years)	Sex (1 = female, 0 = male)	Mean axial length (mm)	Foramen diameter (µm)	IVTS group classification	BCVA logMAR at baseline	BCVA logMar Post surgery	BCVA change	Observational period Post surgery
1	65	0	28.03	790	FTMH: large	1	1	No change	24
2	77	1	27.04	550	FTMH: large	1	0.4	Increase	24
3	83	1	24.47	720	FTMH: large	1.30	0.48	Increase	18
4	69	0	23.36	630	FTMH: large	1.30	0.48	Increase	12
5	70	1	26.21	600	FTMH: large	0.7	0.48	Increase	12
6	69	1	24.05	418	FTMH: large	1	1.8	Decrease	8
7	60	0	33.39	555	FTMH: large	0.4	0.4	No change	5
8	53	1	25.18	671	FTMH: large	0.78	1	Decrease	9
9	63	1	31.30	496	FTMH: large	0.6	0.18	Increase	12
10	80	1	Missing	653	FTMH: large	1	0.3	Increase	6
11	68	1	23.63	567	FTMH: large	1	0.6	Increase	12
12	68	1	22.03	750	FTMH: large	1	0.78	Increase	5
13	70	1	22.50	830	FTMH: large	1	0.6	Increase	2

During surgery, no complications such as local hemorrhage, ILM transplant dislocation or unnecessary traumatization of the central retina occurred.

Anatomical closure of the FTMH was achieved in all 13 patients after surgery. Closure was defined as the re-adaptation of the neurosensory retinal tissue at the rim of the macular hole. Following the classification by Rossi et al., all closures were identified either as Type 1A or Type 1B (Rossi et al., 2020). No cases of disorganization of the retinal inner layers (DRIL) were observed. The observation period ranged from two to 24 months (mean ± SD: 11 ± 7 months, Table 2). During this time, no re-opening of the macular holes occurred in any patient.

**Fig. 1:**
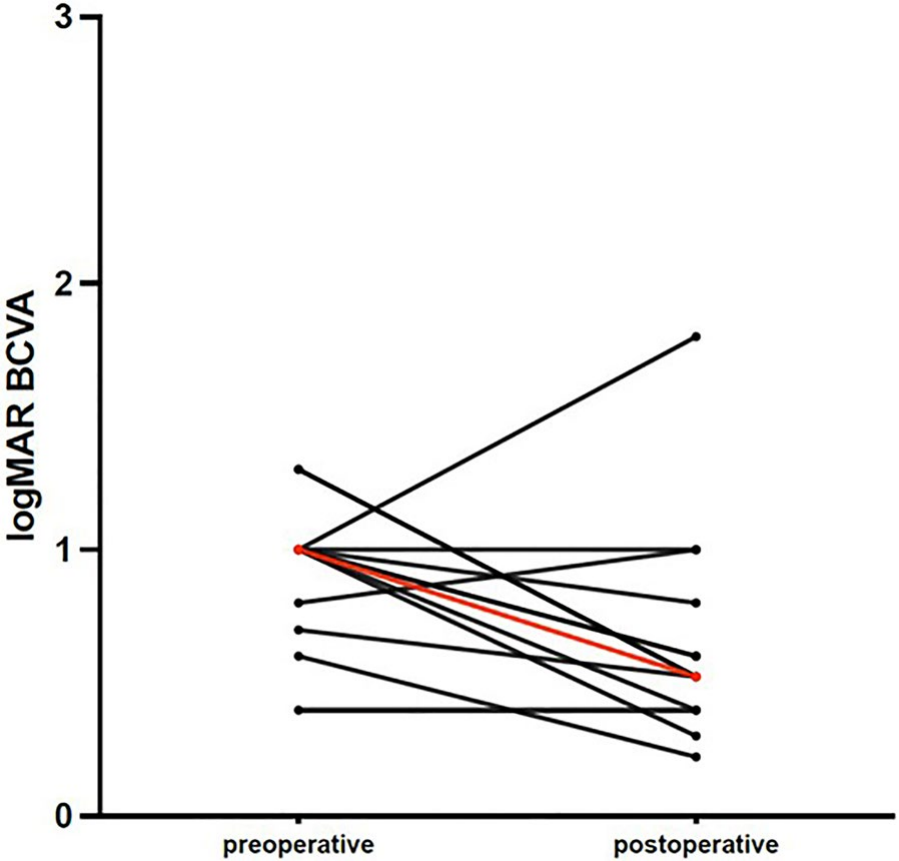
Evolution of pre- versus postoperative BCVA in logMar. In black: individual evolution per patient. In red: average difference

Visual outcomes were also assessed. Pre-operative BCVA logMar ranged from 1.3 to 0.4 (mean ± SD: 0.93 ± 0.25, Table 1). At the last follow-up examination, post-operative BCVA logMar ranged from 1.8 to 0.3 (mean ± SD: 0.66 ± 0.45, Table 1). Overall, 9 out of 13 patients (69.2%) showed significant visual improvement, defined as a gain of one or more Snellen lines, or 0.1 logMAR. In contrast, BCVA remained constant in two patients (15.4%), and vision deteriorated in another two patients (15.4%) due to central macular atrophy (Table 2, Fig. 1).

## Discussion

In our case series of 13 patients, we achieved a 100% rate of anatomical closure of full-thickness macular holes (FTMH) and observed stabilization or significant functional improvement of central best-corrected visual acuity (BCVA) in 84.6% of the patients.

The mean axial length of included eyes was with 27 mm longer than average, which might be due to the fact that myopic FTMH tend to have a reduced closing rate. The eyes observed in this study did not show particular myopic structural changes and no macula demonstrated myopic atrophic changes in the area of the macular hole in advance of the surgery.

Surgery for persistent FTMH that had previous vitrectomies using the gold standard “PPV and ILM peeling” must be discussed in detail with the patient, as the functional results may not be as good as expected by the patient. Nevertheless, Reid et al. concluded in their review questioning the anatomical and functional outcome of re-operation on FTMH that a second surgery achieved a clinically meaningful visual acuity improvement in as much as 58% of patients, with 15% (28 eyes, 13 studies) achieving a BCVA of > 6/12, with an anatomic closure of 78–80% [33].

The authors favor surgery on persistent FTMH and the results of this manuscript are particularly noteworthy when compared to the reported anatomic closure rates for second surgeries on persistent FTMH in existing literature, which range from 47%–85% [13, 15, 17, 19, 34–36].

This article contributes to an ongoing debate in the field about the best surgical method for chronic or persistent FTMH [10].

The presented surgical technique depicts an advancement of previously described techniques. All to date proposed techniques involved placing either autologous or other tissue grafts into or over the FTMH. The described tissues used for this purpose were either autologous free ILM flap [11, 24, 29, 37–40], a lens capsular flap from the anterior or posterior capsule [17, 18], an autologous retinal graft [14, 41] or a human amniotic membrane [13]. All of the grafts were either placed onto the surface of the retina to cover the FTMH or placed inside the FTMH [42]. The authors of this case series see several problems with both techniques:

First, a common problem seen with the graft transplantation to the surface of the retina is intra- or postoperative tissue graft dislocation [29]. To prevent this, some authors have used ocular viscoelastic devices (OVD) or a drop of perfluoro-n-octane to stabilize the ILM flap [11, 39, 43]. Second, Pires et al. analyzed microstructural changes in the fovea after autologous ILM transplant, that was placed inside the FTMH, and were able to show that their closure rate was positively associated with a prolonged proliferation of glial tissue [42]. Thus, the closure might, to some extent, be due to foveal fibrosis. On the contrary, we didn`t find any foveal fibrosis in our cohort. Our surgical technique represents a significant advancement over existing methods, addressing key challenges such as graft dislocation and foveal fibrosis that have been observed with other techniques.

We propose to spread the autologous ILM-transplant subretinal under the rims of the FTMH and expose the outer retina to the transplant. Rizzo et al. transplanted human amniotic membrane in the subretinal space of 8 refractory FTMH and also achieved anatomic and functional success with this method [13]: Rizzo et al. described closure of the FTMH in all eight cases one week after the surgery and reported significantly improved vision. In comparison, our cohort of 13 eyes also demonstrated anatomical restoration in all cases as well as visual improvement. BCVA improved or stabilized in 84.6% (11/13) of patients and 2/13 patients (15.4%) achieved a BCVA of > 0.5.

**Fig. 2:**
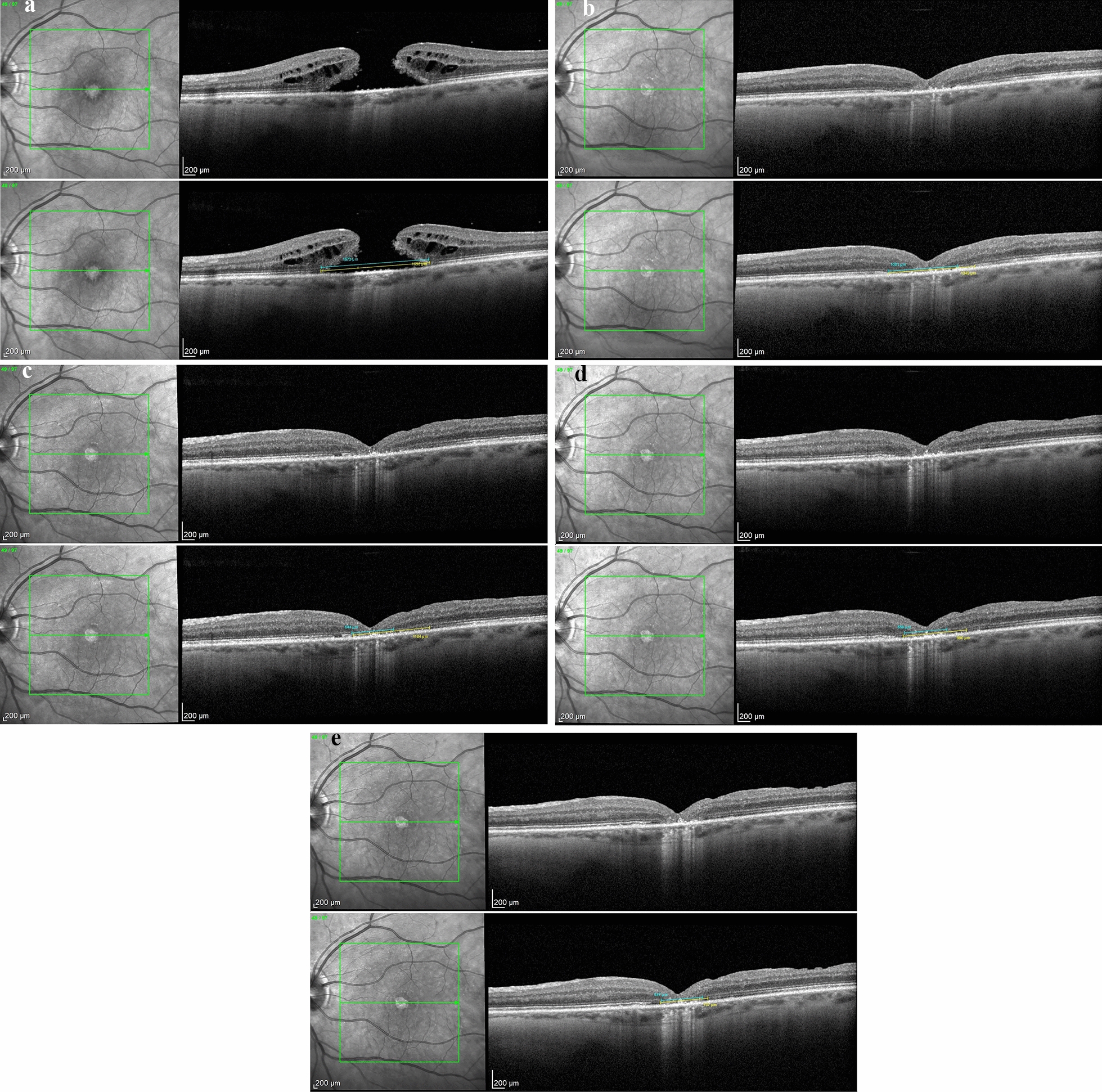
**A**–**E** OCT and BCVA logMar of Patient Nr. 11: preoperative, 1 week, 6 weeks, 6 months and 12 months postoperative. We hypothesize that the external limiting membrane (indicated in blue) and ellipsoid zone (indicated in yellow) are partly being restored over time. **A** Preoperative OCT: BCVA 1.0 logMAR, hole diameter 589 um. **B** 1 week postoperative OCT, BCVA 0.52 logMAR. FTMH closed. **C** 6 weeks postoperative OCT, BVCA 0.6 logMAR. **D** 6 months postoperative, BVCA 0.6 logMAR. **E** 12 months postoperative, BVCA 0.6 logMAR

The idea of both techniques is based on the concept, that a scaffold for the subsequent migration of glial and RPE cells is created. This might induce RPE regeneration and restoration of the outer retina after surgery. Partly restoration of RPE and outer retina in OCT imaging was observed (Fig. 2). With successful FTMH repair, migration of glial and RPE cells may bridge the defect, reestablishing the seal between the neurosensory retina and the RPE gap [44]. Rizzo et al. also reported, that during their follow-up period, the neuroretina differentiated over the human amniotic membrane patch to form retinal layers, in particular the outer layers such as external limiting membrane (ELM) and ellipsoid zone (EZ) [13]. Comparable to this, we also observed partial restoration of the RPE and outer retinal layers, as demonstrated in Fig. 2. Restitution of the RPE could be demonstrated on OCT imaging in all of our patients except the two with central retinal atrophy. An example, where the EZ and ELM defects decreased significantly during the postoperative observation over one year is shown in Fig. 2. BCVA improved from 1.0 logMAR to 0.6 logMAR in this particular patient. This underscores the potential of methods positioning a scaffold in the subretinal space, which might lead to superior structural and functional outcomes after surgery for persistent FTMH.

Two out of 13 patients developed central foveal atrophy after surgery. Both patients have had long-standing large FTMH and underwent multiple previous surgeries. Both corresponding surgeries were uneventful, but special care must be taken when using this maneuver not to manipulate the retina or touch the RPE when placing the ILM under the rim of the hole.

Based on Sano’s postmortem histological studies of closed macular holes, it is suggested that macular holes are closed by proliferative glial cells, which re-approximate the normal photoreceptors to the central fovea. Thus, EZ defects are likely to decrease for several months after surgery. The healing of the EZ varies in closed macular holes, and Sano et al. stated, that since the visual electrical signal is generated in the outer segments of the photoreceptors, the visual outcome must be related to the integrity of the EZ in closed macular holes [45].

Elhusseiny et al. published a 10-year-follow-up observation of the BCVA after FTMH surgery and demonstrated, that visual acuity improvement after FTMH surgery continued during the first 3 years after surgery and that improvement in the postoperative BCVA remained stable 10 years after surgery [46]. Therefore, it may be hypothesized that functional and anatomic outcomes may further improve in individual patients of this case series.

Regarding functional restoration, microperimetry measurements would be desirable regarding the structure–function-correlated outcome.

We suggest a limited functional restoration of photoreceptors, since RPE, ELM and EZ tend to regenerate towards the center of the fovea in the postoperative observation period (Fig. 2).

## Conclusion

In this article, we introduce a novel surgical technique for treating persistent FTMH. Our case series demonstrates that reoperation placing autologous ILM in the subretinal space achieves a 100% success rate and shows promising functional outcomes.

However, this case-cohort study has several limitations. Firstly, the cases were studied retrospectively and a randomized evaluation of the surgical method would be desirable. Secondly, further studies with more sophisticated functional testing (e.g. using microperimetry) and a longer follow-up period would be helpful to evaluate, whether the macular hole is closed by functional retinal tissue or by scar tissue.

### Supplementary Information

**Additional file 1: Video S1.** Example of surgical management of the presented surgical technique using a free ILM flap and spreading it subretinal under the rims of the FTMH.

## Data Availability

Most data generated or analysed during this study are included in this published article. Any not included datasets used and/or analysed during the current study are available from the corresponding author on reasonable request.
